# Identification of Loop D Domain Amino Acids in the Human Aquaporin-1 Channel Involved in Activation of the Ionic Conductance and Inhibition by AqB011

**DOI:** 10.3389/fchem.2018.00142

**Published:** 2018-04-27

**Authors:** Mohamad Kourghi, Michael L. De Ieso, Saeed Nourmohammadi, Jinxin V. Pei, Andrea J. Yool

**Affiliations:** Aquaporin Physiology and Drug Discovery Program, Adelaide Medical School, University of Adelaide, Adelaide, SA, Australia

**Keywords:** major intrinsic protein, MIP, AQP1, water channel, non-selective cation channel, cyclic GMP, arylsulfonamide

## Abstract

Aquaporins are integral proteins that facilitate the transmembrane transport of water and small solutes. In addition to enabling water flux, mammalian Aquaporin-1 (AQP1) channels activated by cyclic GMP can carry non-selective monovalent cation currents, selectively blocked by arylsulfonamide compounds AqB007 (IC_50_ 170 μM) and AqB011 (IC_50_ 14 μM). *In silico* models suggested that ligand docking might involve the cytoplasmic loop D (between AQP1 transmembrane domains 4 and 5), but the predicted site of interaction remained to be tested. Work here shows that mutagenesis of two conserved arginine residues in loop D slowed the activation of the AQP1 ion conductance and impaired the sensitivity of the channel to block by AqB011. Substitution of residues in loop D with proline showed effects on ion conductance amplitude that varied with position, suggesting that the structural conformation of loop D is important for AQP1 channel gating. Human AQP1 wild type, AQP1 mutant channels with alanines substituted for two arginines (R159A+R160A), and mutants with proline substituted for single residues threonine (T157P), aspartate (D158P), arginine (R159P, R160P), or glycine (G165P) were expressed in *Xenopus laevis* oocytes. Conductance responses were analyzed by two-electrode voltage clamp. Optical osmotic swelling assays and confocal microscopy were used to confirm mutant and wild type AQP1-expressing oocytes were expressed in the plasma membrane. After application of membrane-permeable cGMP, R159A+R160A channels had a significantly slower rate of activation as compared with wild type, consistent with impaired gating. AQP1 R159A+R160A channels showed no significant block by AqB011 at 50 μM, in contrast to the wild type channel which was blocked effectively. T157P, D158P, and R160P mutations had impaired activation compared to wild type; R159P showed no significant effect; and G165P appeared to augment the conductance amplitude. These findings provide evidence for the role of the loop D as a gating domain for AQP1 ion channels, and identify the likely site of interaction of AqB011 in the proximal loop D sequence.

## Introduction

Aquaporins (AQPs) are a diverse family of channels for water and solutes, classified as major intrinsic proteins (MIPs) (Benga et al., 1986; Agre et al., 1993; Reizer et al., 1993). In mammals, classes of AQPs are differentially expressed in endothelial, epithelial and other cell types, and comprise key components of mechanisms for fluid homeostasis in single cells, barrier tissues, and organs (Nielsen et al., 1993; Boassa and Yool, 2005; Hachez and Chaumont, 2010). Some classes of aquaporin channels have been found shown to transport molecules other than water across the cell membrane, including glycerol, ammonia, urea, protons, as well as CO_2_ and O_2_ gases (Madeira et al., 2014; Kitchen et al., 2015), and ions (Yool, 2007; Yool and Campbell, 2012).

Aquaporin ion channel functions have been described for multiple members of the MIP family. Recent work has shown that a plant aquaporin channel (AtPIP2;1) serves as a non-selective cation channel that is sensitive to Ca^2+^ and pH (Byrt et al., 2016), addressing a mystery regarding the molecular basis of a Ca^2+^-inhibited leak current known to be involved in environmental stress responses of roots (Demidchik and Tester, 2002). The insect aquaporin Big Brain (BIB) channel in *Drosophila* (Yanochko and Yool, 2002) and mammalian lens MIP (AQP0) have been characterized as ion channels (Zampighi et al., 1985; Ehring et al., 1990); their importance of these channels is evident from the consequences of genetic knockouts resulting in impaired nervous system development (Rao et al., 1992) and cataract formation (Berry et al., 2000), respectively. However the precise roles of their ion channel activities in cell signaling and development remain to be determined.

Controversy on the role of AQP1 as an ion channel, first proposed in 1996 (Yool et al., 1996), stemmed from a paradigm which stated AQP1 was nothing but a water channel (Tsunoda et al., 2004). An extensive body of work published since has shown: (i) AQP1 is a dual water and cation channel with a unitary conductance of 150 pS under physiological conditions, permeable to Na^+^, K^+^, and Cs^+^, and gated by the binding of cGMP at the intracellular loop D domain (Anthony et al., 2000; Yu et al., 2006). (ii) AQP1 carries water through the individual intra-subunit pores, whereas cations pass through the central pore of the tetramer (Yu et al., 2006; Campbell et al., 2012). (iii) Single channel activity of natively expressed AQP1 is selectively lost after small interfering knockdown of AQP1 expression (Boassa et al., 2006). (iv) The availability of AQP1 to be activated as an ion channel is regulated by tyrosine kinase phosphorylation of the carboxyl terminal domain (Campbell et al., 2012). (v) AQP1 ion channel properties are altered by site-directed mutagenesis of the central pore domain, which changes the cationic selectivity of the current, and creates a gain-of-function blocking site by Hg^2+^ via introduction of a cysteine residue at the extracellular side (Campbell et al., 2012). (vi) Mutations of the carboxyl terminal domain of hAQP1 alter the efficacy of cGMP in activating the ionic conductance (Boassa and Yool, 2003). (vii) Molecular dynamic simulations confirmed it was theoretically feasible to move Na^+^ ions through the AQP1 central pore and identified the cytoplasmic loop D domain as involved in gating of the ion channel; mutation of key loop D residues impaired ion channel activation without preventing water channel activity (Yu et al., 2006).

The ability to change specific ion channel properties of activation, ion selectivity, and block using site-directed mutations of the AQP1 amino acid sequence have provided convincing evidence that AQP1 directly mediates the observed ionic current (Anthony et al., 2000; Boassa and Yool, 2003; Yu et al., 2006; Campbell et al., 2012). The alternative suggestion that responses were due to unidentified native ion channels translocated into the membrane along with AQP1 was ruled out by these studies, which showed that the altered ion channel functions associated with mutations of AQP1 did not prevent normal assembly and plasma membrane expression of AQP1 channels as evidenced by immunolabeling, western blot, and measures of osmotic water permeability.

While the ion channel function of AQP1 was confirmed independently by other groups (Saparov et al., 2001; Zhang et al., 2007), the physiological relevance of AQP1 ion channel function remained uncertain, given the low proportion of ion conducting channels observed in reconstituted membrane assays. Mathematical modeling tested the premise, assuming only a tiny fraction of AQP1 acted as ion channels, and showed the predicted effects were sufficient for a meaningful impact on net transport in epithelial cells (Yool and Weinstein, 2002). Interestingly the relative amplitudes of ion currents and water fluxes for mammalian AQP6, also thought to be a dual water and ion channel (Yasui et al., 1999), were similar to those of AQP1, suggesting AQP6 similarly has a low proportion of functioning ion channels within the total population. Although high densities of water channels might be needed to move substantial fluid volumes, the apparently low ratios for aquaporins reinforce a basic concept in the ion channel field; relatively few charge-selective ion channels are needed to alter transmembrane voltage gradients (Hille, 2001).

With development of the first selective AQP1 ion channel inhibitor AqB011 (Kourghi et al., 2016), the question of the physiological function of the AQP1 ion channel could be directly addressed. Kourghi and colleagues showed AqB011 selectively inhibited migration in AQP1-expressing cancer cell lines, but not in those without AQP1, demonstrating that the AQP1 ion conductance can serve an essential role in cellular functions such as migration. Of the pharmacological inhibitors of AQP1 ion channel identified thus far, AqB011 is the most potent (IC_50_ 14 μM). Osmotic water fluxes in hAQP1-expressing oocytes were not altered by 200 μM AqB011, indicating the block is selective for AQP1 ion channel activity. Molecular docking models suggested loop D domain as a candidate binding site for the AqB011 (Kourghi et al., 2016), but the prediction remained to be tested.

The role of AQP1 loop D residues in ion conductance activation and in mediating block by AqB011 was tested here using site-directed mutations of amino acids. Conserved arginine residues at positions 159 and 160 in human AQP1 were mutated to alanines. As compared with wild type, the cGMP-mediated activation of the AQP1 ionic conductance response was significantly slower in R159A+R160A channels, the maximal amplitude of the activated current in the mutant construct was reduced as compared to wild type, and the mutant was insensitive to the inhibitor AqB011. Human AQP1 mutant constructs in which proline was substituted for conserved single residues threonine (T157P), aspartate (D158P), arginine (R159P, R160P), and glycine (G165P) showed differential effects on conductance activation depending on position, which suggested the conformation of loop D is important for AQP1 ion channel gating. Proline enables tight bends in peptide structures (Vanhoof et al., 1995). These results support the role of conserved loop D residues in AQP1 ion channel activation and inhibition by AqB011, and provide further support for the concept that loop D is a gating domain for the AQP1 central ion pore.

## Materials and methods

### Oocyte preparation and injection

Unfertilized oocytes were harvested by partial ovariectomy from anesthetized *Xenopus laevis* frogs following national guidelines (Australian Code of Practice for the Care and Use of Animals for Scientific Purposes), and approved by the University of Adelaide Animal Ethics Committee (approval # M2013-167). Oocytes were defolliculated with collagenase (type 1A, 1 mg/ml; Sigma-Aldrich, St. Louis, MO) in the presence of trypsin inhibitor (0.05 mg/ml; Sigma-Aldrich, St. Louis, MO) for 1 to 1.5 h in OR-2 saline (96 mM NaCl, 2 mM KCl, 5 mM MgCl_2_, penicillin 100 units/ml, streptomycin 0.1 mg/ml, and 5 mM HEPES; pH 7.6). Oocytes were then washed 4 times with OR-2 saline at ~10 min intervals, and kept at 16–18°C in isotonic Frog Ringers saline [96 mM NaCl, 2 mM KCl, 5 mM MgCl_2_, 0.6 mM CaCl_2_, 5 mM HEPES buffer, horse serum (5%; Sigma-Aldrich, St. Louis, MO), penicillin 100 units/ml streptomycin 0.1 mg/ml, and tetracycline 0.5 mg/ml, pH 7.6]. Oocytes were injected with 50 nl of water (control oocytes), or 50 nl of water containing 1 ng of AQP1 wild type cRNA, or 2 ng of AQP1 mutant cRNAs. Oocytes were then transferred to sterile dishes containing Frog Ringers saline and incubated at 16–18°C for 48 h or more to allow time for protein expression. Isotonic Na^+^ saline used for electrophysiology and osmotic swelling assays contained (in mM): NaCl 96 mM, KCl 2 mM, MgCl_2_ 5 mM, CaCl_2_ 0.6 mM, and HEPES 5 mM, pH 7.3, without antibiotics or serum.

### Site directed mutagenesis of AQP1

Site-directed mutations were generated in human AQP1 cDNA in the *Xenopus* expression vector (pxBGev), using the QuikChange site-directed mutagenesis kit (Agilent Technologies, Forest Hills, VIC, Australia) with custom-synthesized primers as described previously (Yu et al., 2006). The correct sequences of the constructs were confirmed by replicate DNA sequencing of the full-length cDNA constructs. Wild-type AQP1 and mutant cDNAs were linearized using BamHI and transcribed with T3 RNA polymerase using the mMessage mMachine kit (Ambion, Austin, TX).

### Osmotic swelling assays and confocal microscopy

Swelling assays or confocal microscopy were used to confirm AQP1 wild type and mutant channels were expressed in oocyte plasma membranes. Swelling assays were performed in 50% hypotonic saline (isotonic Na^+^ saline diluted with equal volume of water). Prior to swelling assays the control (non-AQP expressing), AQP1 wild type and AQP1 mutant expressing oocytes were rinsed in isotonic saline (without horse serum or antibiotics) for 10 min. Rates of swelling were imaged using a grayscale camera (Cohu, San Diego, CA) fixed on a dissecting microscope (Olympus SZ-PT; Olympus, Macquarie Park, Australia), and images were captured at 0.5 Hz using Image J software from National Institutes of Health (http://rsbweb.nih.gov/ij/). Swelling rates were determined from slope values of linear regression fits of relative volume as a function of time using Prism (GraphPad Software Inc., San Diego, CA). For confocal microscopy, oocytes were fixed in 4% paraformaldehyde, permeabilized with 0.1% Triton X-100, and incubated with rabbit polyclonal anti-AQP1 antibody (provided by WD Stamer; Duke University, USA) diluted in buffered solution with 300 mM NaCl, 30 mM Na citrate, 1% bovine serum albumin, 0.05% TritonX-100, and 0.02% sodium azide. After secondary labeling with FITC-conjugated goat anti-rat antibody, preparations were imaged with a Leica (Nussloch, Germany) TCS-4D laser scanning confocal microscope.

### Electrophysiological recordings

Two-electrode voltage clamp recordings were performed at room temperature in standard isotonic Na^+^ saline using a GeneClamp amplifier and Clampex 9.0 software (pClamp 9.0 Molecular Devices, Sunnyvale, CA, USA). Data were filtered at 2 kHz and stored to hard disk for analysis. Capillary glass pipettes (~1 MΩ) were filled with 1 M KCl. Initial conductance values were determined from current-voltage relationships measured prior to cGMP stimulation, by application of the nitric oxide donor sodium nitroprusside (SNP) at a final concentration of 7.5 mM, or by application of membrane permeable CPT-cGMP(8-(4-chlorophenylthio)-guanosine-3′,5′-cyclic monophosphate) at a final concentration of 10 μM, as per published methods (Boassa and Yool, 2003; Campbell et al., 2012). From a holding potential of −40, voltage steps from +60 to −110 mV were applied to measure conductance. Repeated steps to +40 mV at 6 s intervals were used to monitor changes in ion current responses as a function of time after application of an activator or inhibitor. For the studies of pharmacological inhibition by AqB011, after recording the conductance for the first response to CPT-cGMP, oocytes were transferred into isotonic Na^+^ saline with either AqB011 or vehicle for 2 h. Incubation allowed recovery to initial conductance levels as well as time for AqB011 to cross the membrane to reach its intracellular site of action, as described previously (Kourghi et al., 2016). Recovery from block was very slow, taking hours after removal of the agent from the extracellular medium. Oocytes were then re-evaluated for responsiveness to a second application of CPT-cGMP to test for inhibition post-incubation without AqB011 present. AqB011 was synthesized by G Flynn (SpaceFill Enterprises LLC, Bozeman Montana USA) with preparation methods and chemical structure as previously published (Kourghi et al., 2016). AqB011 was prepared as a 1000x stock solution in the vehicle dimethylsulfoxide (DMSO) and diluted in recording saline to the final concentration; vehicle control saline was made with the equivalent amount of DMSO (0.1% V/V). Box plot histograms show 50% of data (boxes), the full range of data (error bars), and the median value (horizontal bar).

## Results

### Reduced sensitivity to block by AqB011 in AQP1 R159A+R160A channels

**Figure 1:**
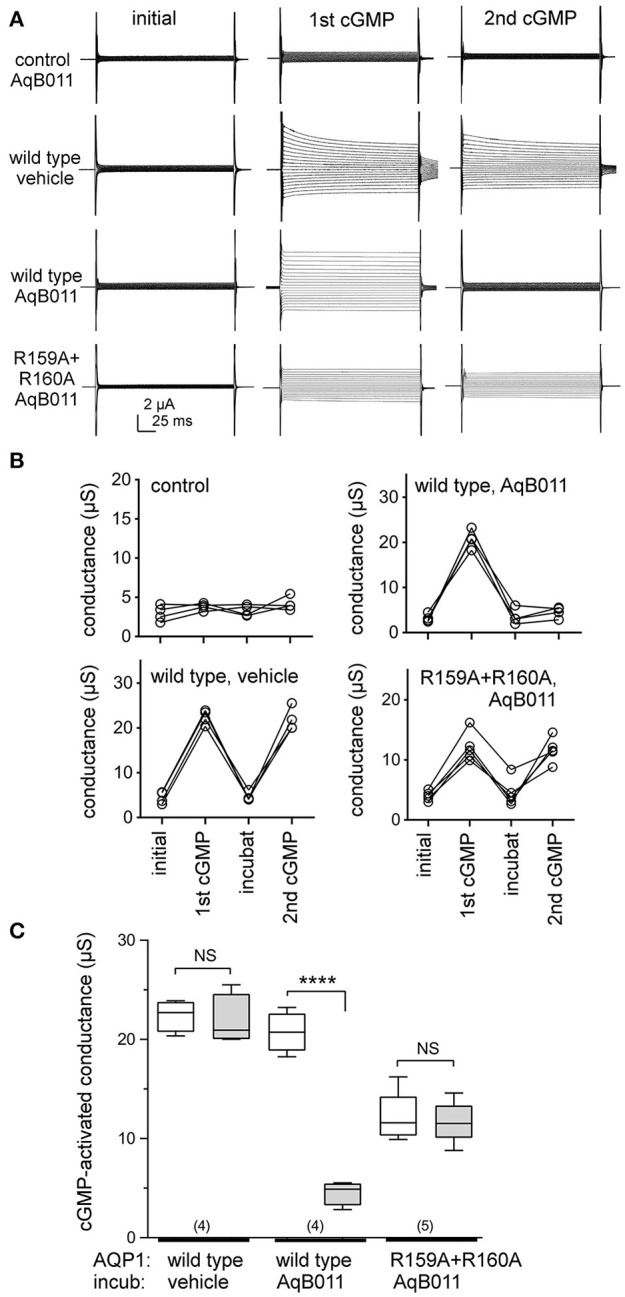
Human AQP1 ionic conductances activated by cGMP differ in sensitivity to the inhibitor AqB011 in wild type and R159A+R160A expressing oocytes. **(A)** Electrophysiology traces showing currents recorded in control non-AQP oocytes, and in hAQP1 wild type and R159A+R160A expressing oocytes. The current traces are shown prior to stimulation (initial), after the first maximal response to CPT-cGMP (1st cGMP), and after the second maximal response (2nd cGMP) following a 2 h incubation with 50 μM AqB011 or vehicle (DMSO). **(B)** Trend plots show the ionic conductance amplitudes for individual oocytes through each series of treatments for AQP1 wild type, mutant, and non AQP-expressing control oocytes, measured before stimulation (initial), after the first CPT-cGMP (1st cGMP), after 2-h recovery in cGMP-free saline containing vehicle or 50 μM AqB011 (“incubat”), and after the second CPT-cGMP (2nd cGMP). **(C)** Compiled box plot data illustrate statistically significant block of AQP1 wild type but not R159A+R160 ion conductances following incubation in 50 μM AqB011. *n* values are above the x-axis. Boxes show 50% of data points; error bars show the full range; horizontal bars show median values. ^****^p < 0.0001.

Voltage clamp recordings showed that application of extracellular CPT-cGMP activated ionic conductance responses in human AQP1 wild type and R159A+R160A mutant expressing oocytes (Figure 1A). Initial recordings measured before the application of CPT-cGMP showed uniformly low currents, comparable to those of non-AQP control oocytes. The ionic conductance increased after application of CPT-cGMP in AQP1 wild type and R159A+R160A expressing oocytes, but in not non-AQP control oocytes. After recording the first response, oocytes were transferred into isotonic Na^+^ saline with 50 μM AqB011 or vehicle. Figure 1B shows trend plots of the conductance responses of individual oocytes through each series of treatments. After 2 h incubation, the ionic conductance responses recovered to initial levels, and a second application of CPT-cGMP was used to assess the level of reactivation of current (Figures 1A,B). CPT-cGMP activated currents were not observed in non-AQP expressing control oocytes. Figure 1C shows compiled box plot data for the ionic conductance values for human AQP1 wild type and R159A+R160A mutants. AQP1 wild type currents were strongly blocked after incubation in AqB011 but not after incubation with vehicle. The amplitude of maximal activation was lower in R159A+R160A mutant-expressing oocytes than wild type, and the R159A+R160A conductance was not sensitive to block by AqB011.

The recovery of the AQP1 wild type and mutant currents to baseline levels during the incubation period demonstrated that the responses were reversible, thus not due to oocyte damage or leak. Complete reactivation of wild type ionic conductance response to the second application of CPT-cGMP (after incubation in saline with vehicle) demonstrated that prior activation did not impair responsiveness of the AQP1-expressing oocytes to subsequent stimulation. AQP1 wild type-expressing oocytes incubated in saline with AqB011 were not re-activated by a second application of CPT-cGMP, confirming inhibition of the ion current as described previously (Kourghi et al., 2016). In contrast, the AQP1 R159A+R160A mutant channels showed no change in the second response to CPT-cGMP after incubation with or without AqB011, showing that sensitivity to the inhibitor was eliminated by the altered loop D sequence. The insensitivity of the R159A+R160A current furthermore demonstrated that the observed pharmacological block of wild type current by AqB011 cannot readily be ascribed to off-target effects on native oocyte channels or transporters, confirming the specificity of action of the antagonist compound.

### Increased latency to activation for AQP1 R159A+R160A channels

**Figure 2:**
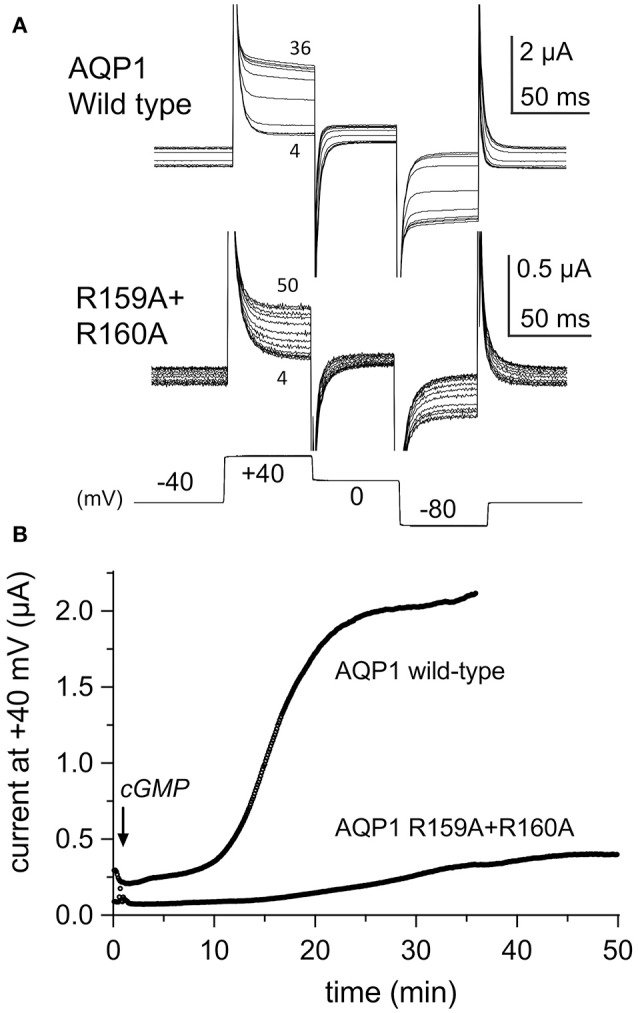
Rates of activation of ion conductance responses to CPT-cGMP in oocytes expressing AQP1 wild type or R159A+R160A channels. **(A)** Ion current responses were monitored after application of CPT-cGMP using repeated series of brief steps to +40, 0, and −80 mV from a holding potential of −40 mV (10 per minute; 150 ms each). Traces are shown at 4 min intervals for clarity. Numbers indicate time in minutes post-application of CPT-cGMP. **(B)** The plot of steady state current amplitudes at +40 mV as a function of time after application of CPT-cGMP at time zero illustrates the difference in latency to activation in representative examples of AQP1 wild type and R159A+R160A expressing oocytes.

The conductance responses of wild type and R159A+R160A mutant channels differed in rates of activation after application of CPT-cGMP. Oocytes expressing AQP1 wild type activated more rapidly and reached a higher maximal current amplitude that did those expressing AQP1 R159A+R160A channels (Figure 2A). In wild type, the maximal response was reached by ~20–30 min after application of CPT-cGMP, whereas 50–60 min was needed for R159A+R160A expressing oocytes (Figure 2B). The latency to the onset of activation was considerably slower for the mutant construct. The long latency for R159A+R160A was consistent with prior work which reported no appreciable activation of the R159A+R160A mutant channels when assessed over a short time frame (within 8 min after application of the nitric oxide donor, sodium nitroprusside, which was used to stimulate endogenous oocyte cGMP production, and successfully activated AQP1 wild type ion currents) (Yu et al., 2006).

### Osmotic water permeability of AQP1 wild type and R159A+R160A expressing oocytes

**Figure 3:**
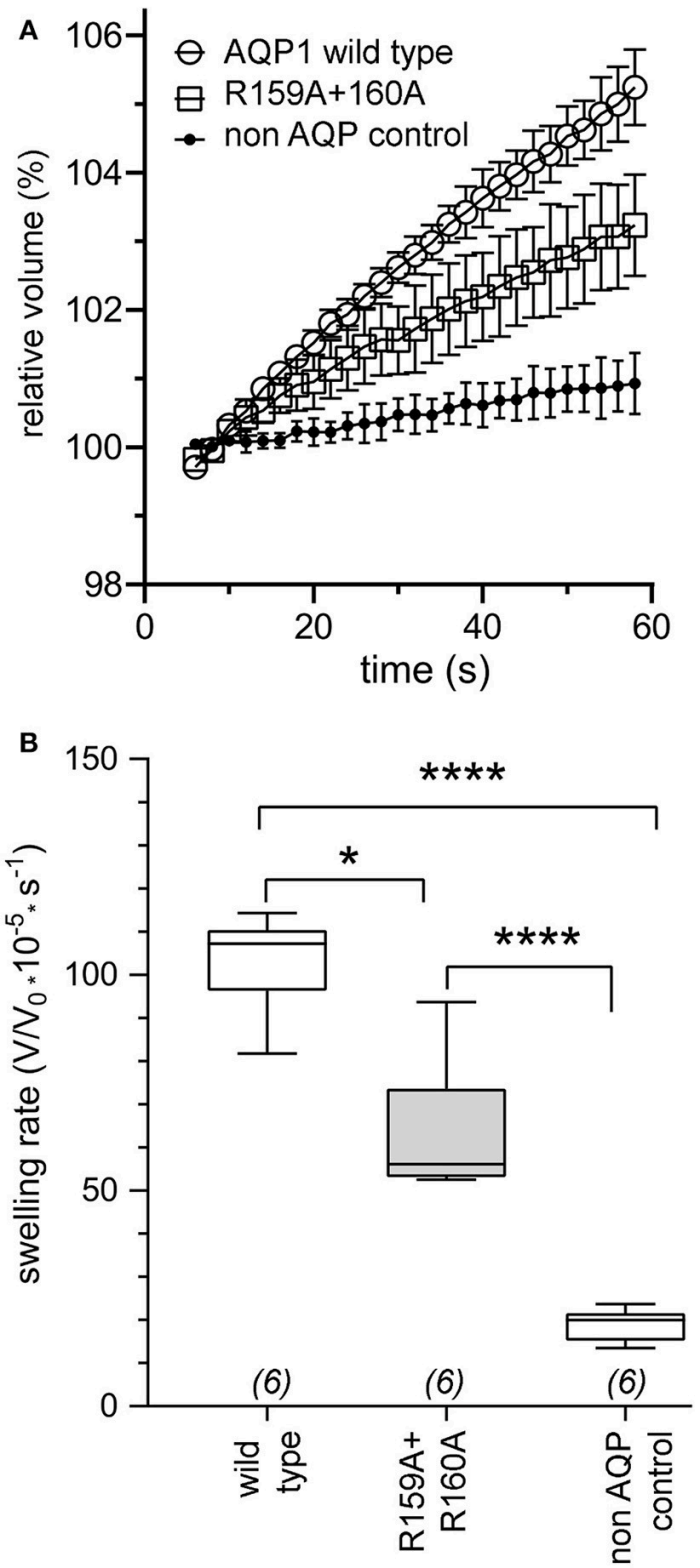
Confirmation of expression of AQP1 wild type and AQP1 R159A+R160 mutant channels in oocyte plasma membranes by significantly increased osmotic water permeabilities as compared to non-AQP1 expressing controls. **(A)** Osmotic water permeabilities (mean ± SEM) assessed by quantitative swelling assays for AQP1 wild type (open circles) and AQP1 R159A+R160 mutant (squares) compared with non-AQP1 expressing control oocytes (filled circles). Relative volumes as a function of time after introduction into 50% hypotonic saline at time zero were measured from video-imaged cross-sectional areas. (*n* = 6 per group). **(B)** Box plot data showing osmotic swelling rates were higher in oocytes expressing AQP1 wild type and AQP1 R159A+R160 mutants than non-AQP1 expressing controls (one way ANOVA; *post-hoc* Bonferroni tests). ^*^p < 0.05; ^****^p < 0.0001; *n* = 6 per group. Boxes show 50% of data points; error bars show the full range; horizontal bars show median values.

Osmotic water permeability data (Figure 3A) confirmed successful expression of wild type and R159A+R160A mutant AQP1 channels in oocyte plasma membranes. The water channel activities of AQP1 wild type and R159A+R160A expressing oocytes were both were significantly greater than those of non-AQP1 expressing control oocytes (Figure 3B), confirming that both AQP1 channel types were expressed, assembled, and trafficked to the plasma membrane of oocytes. Expression levels for the R159A+R160A mutant channels estimated by osmotic water permeability were ~10% lower than wild type; however the mean conductance response in the arginine double mutant (Figure 1C) was half that of wild type, consistent with impairment of channel activation.

### Effects of proline mutagenesis of the loop D amino acid sequence

**Figure 4:**
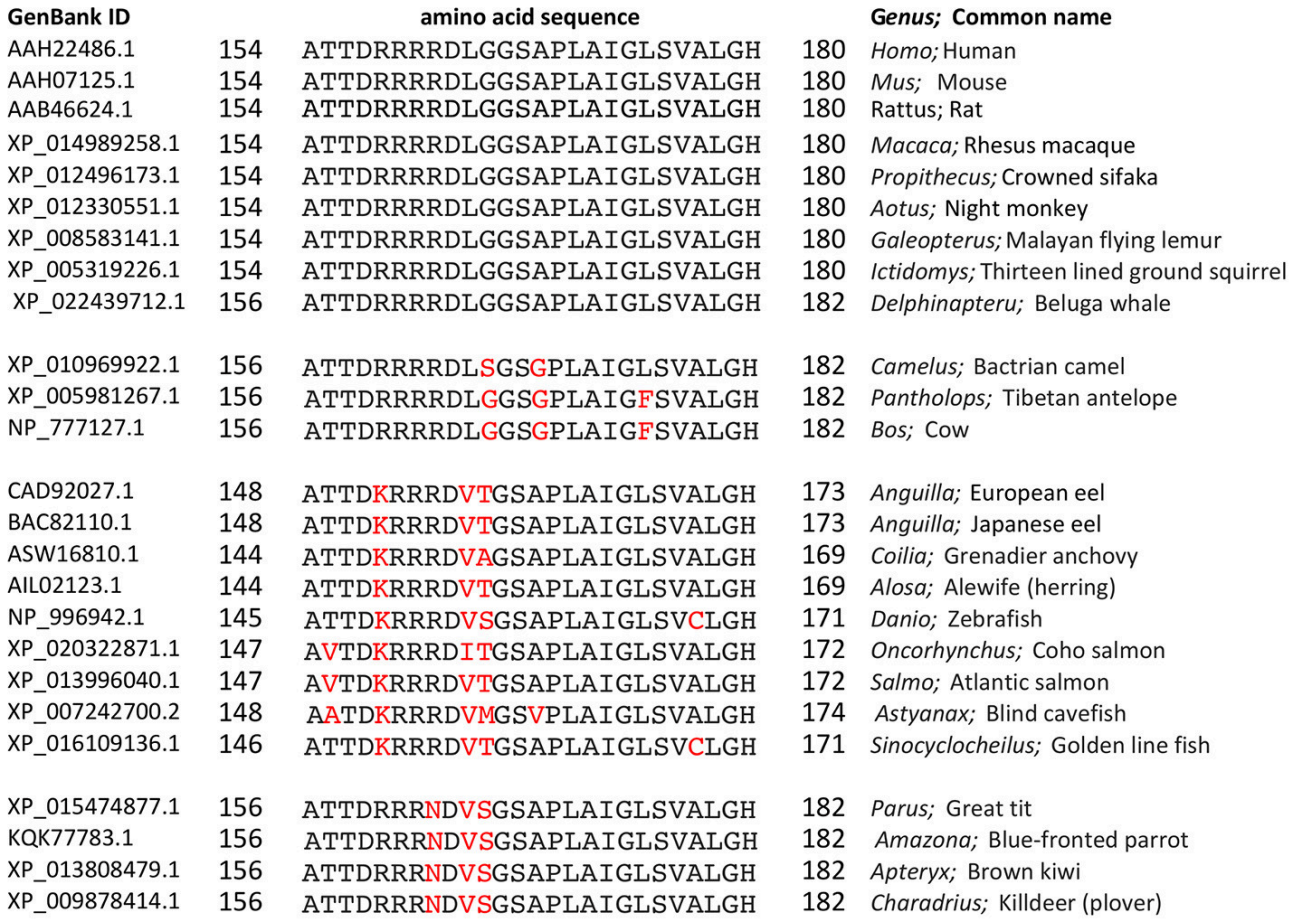
Amino acid sequence alignment for the loop D and flanking domains of AQP1 channels from diverse classes of vertebrates (mammals, fish, and birds). Amino acid sequences downloaded from the National Center for Biotechnology Information (NCBI) Protein database (www.ncbi.nlm.nih.gov/protein) were aligned using the NCBI BlastP online application (blast.ncbi.nlm.nih.gov) for multiple sequences. Residues in black are identical with the query sequence *Homo sapiens* AQP1. Variations in sequence are highlighted in red.

**Figure 5:**
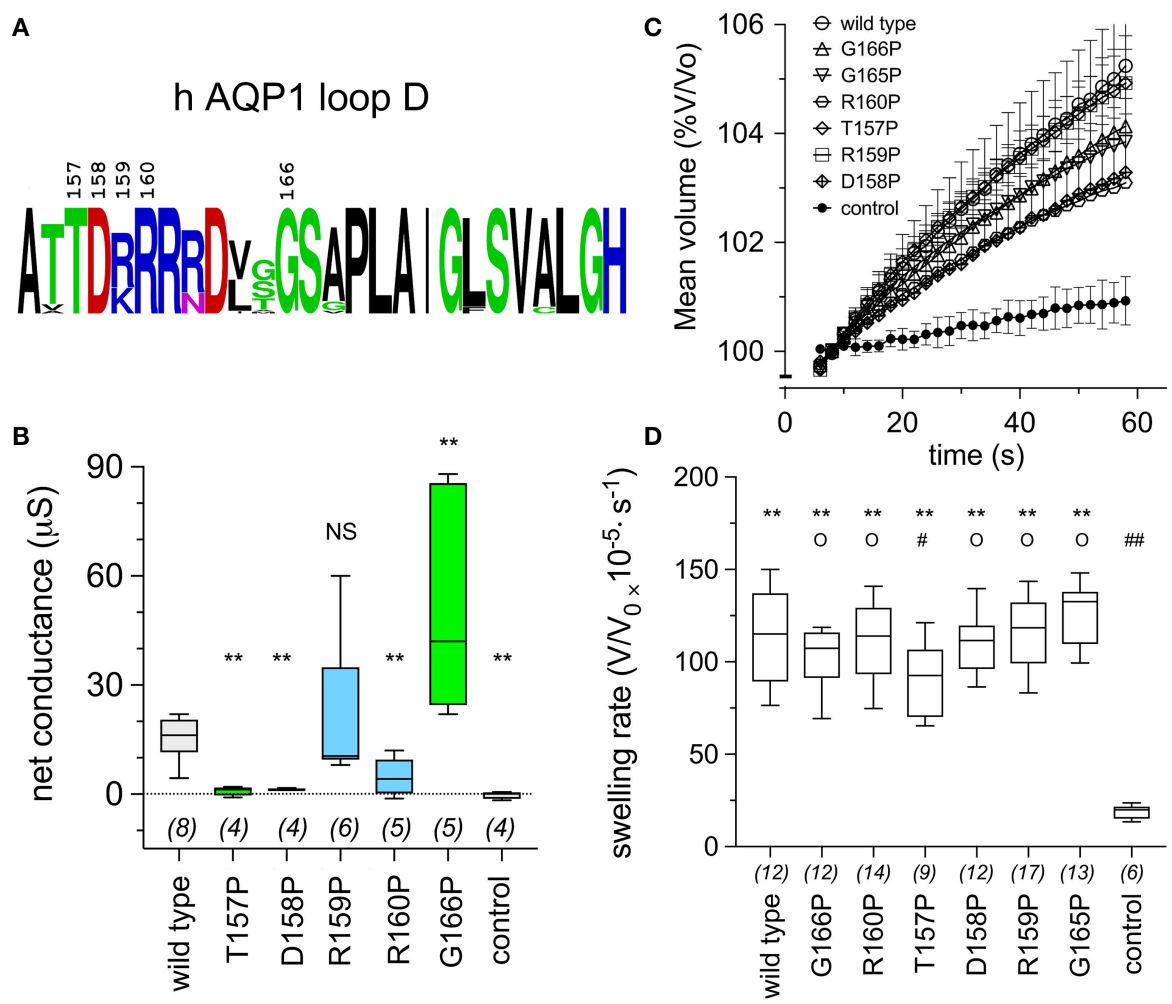
Conserved amino acids in the AQP1 loop D domain influence the ion conductance response, as assessed by proline mutagenesis. **(A)** Schematic summary of the level of conservation of loop D amino acids in AQP1 sequences (data shown in Figure 4) created with the online WebLogo tool (http://weblogo.berkeley.edu/logo.cgi). Letter sizes represent corresponding relative frequencies of occurrence at that position in the sequence set. **(B)** Box plot data showing the net conductance values (maximal—initial) for hAQP1 wild type and proline substituted mutant channels. Position-specific effects of proline-scanning mutagenesis on ion conductance responses in human AQP1 suggested less conserved positions are more tolerant of proline substitution. Statistical significance was evaluated with ANOVA and *post-hoc* two-tailed Mann Whitney. ^**^p < 0.01; NS not significant, as compared with wild type; *n* values are above the x-axis. **(C)** Graph of mean volumes measured during optical swelling assays and standardized as a percentage of initial volume, for control oocytes and oocytes expressing wild type hAQP1 and proline-substituted mutants as a function of time after introduction of the oocyte into 50% hypotonic saline at time zero. (*n* values given in **D**). **(D)** Box plot histogram of swelling rates for hAQP1 wild type, proline mutant and control oocytes. Significant differences were determined by ANOVA (*p* < 0.0001) and *post-hoc* unpaired *T*-tests. ^**^ indicates a significant difference from control, *p* < 0.0001. O indicates no significant difference from wild type, *p* > 0.05. A significant difference from wild type is indicated as # at *p* < 0.05, or ## for *p* < 0.0001.

**Figure 6:**
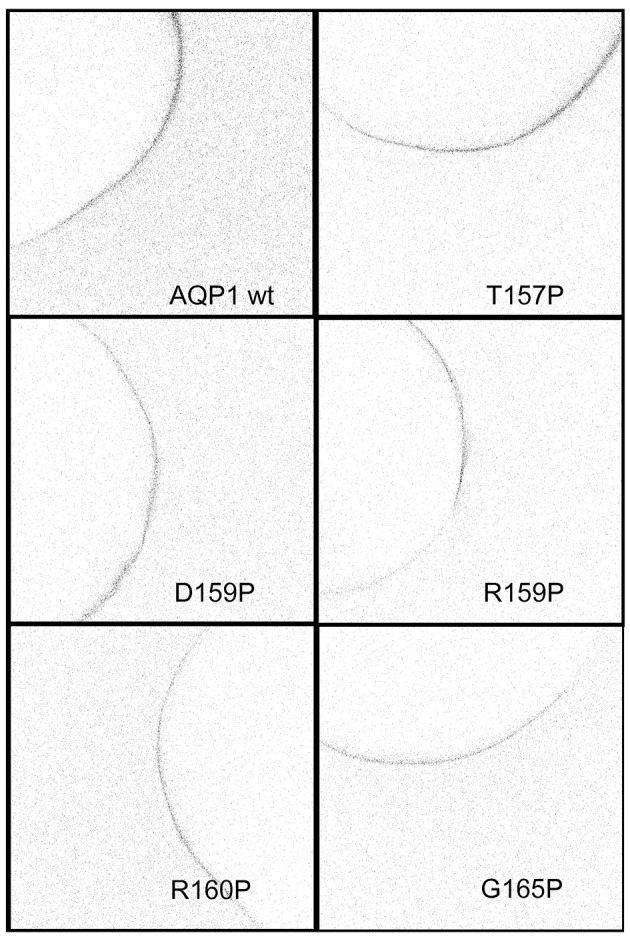
Confocal images of anti-AQP1 immunolabeled oocytes expressing wild type and proline substituted mutant channels confirmed protein expression in the oocyte plasma membrane. See Methods for details.

Proline substituted mutant channels showed significant differences in response amplitudes that correlated with the degree of conservation of the amino acid residue in the loop D sequence. Sequence alignments for loop D and flanking domains illustrate the high level of identity for amino acids in AQP1 gene coding sequences from a diverse array of vertebrates, including mammals, fish, and birds (Figure 4). Net conductances, measured from amplitudes of the ionic conductance response, were calculated as the difference between the initial level and the final amplitude after SNP-mediated cGMP stimulation (Figure 5). Wild type AQP1 channels showed activation in response to SNP stimulation (Figure 5B) that was comparable in amplitude to that seen after application of CPT-cGMP (Figure 1). Control non-AQP-expressing oocytes showed no appreciable response. However, significantly impaired responses were seen for oocytes expressing AQP1 T157P, D158P, and R160P mutant channels (Figure 5B). T157, D158, and R160 residues exhibit complete identity across AQP1 sequences from diverse animals (Figure 5A). In contrast, AQP1 R159P expressing oocytes showed no significant difference from AQP1 wild type, which could fit with the observation that slightly more variation in amino acid sequence appears to be tolerated at that position. Interestingly, a significant difference also was observed for mutation to proline at the highly conserved G166, but the result was to promote rather than inhibit the activation of the conductance response as compared to wild type. The expression of functional channels in the oocyte membrane was confirmed by the demonstration of high osmotic water permeabilities for all the proline mutant constructs that were significantly greater that that of the non-AQP-expressing controls (Figures 5C,D). Confocal images of oocytes expressing AQP1 wild type and proline mutant channels confirmed that the constructs were expressed in the oocyte plasma membrane (Figure 6).

**Figure 7:**
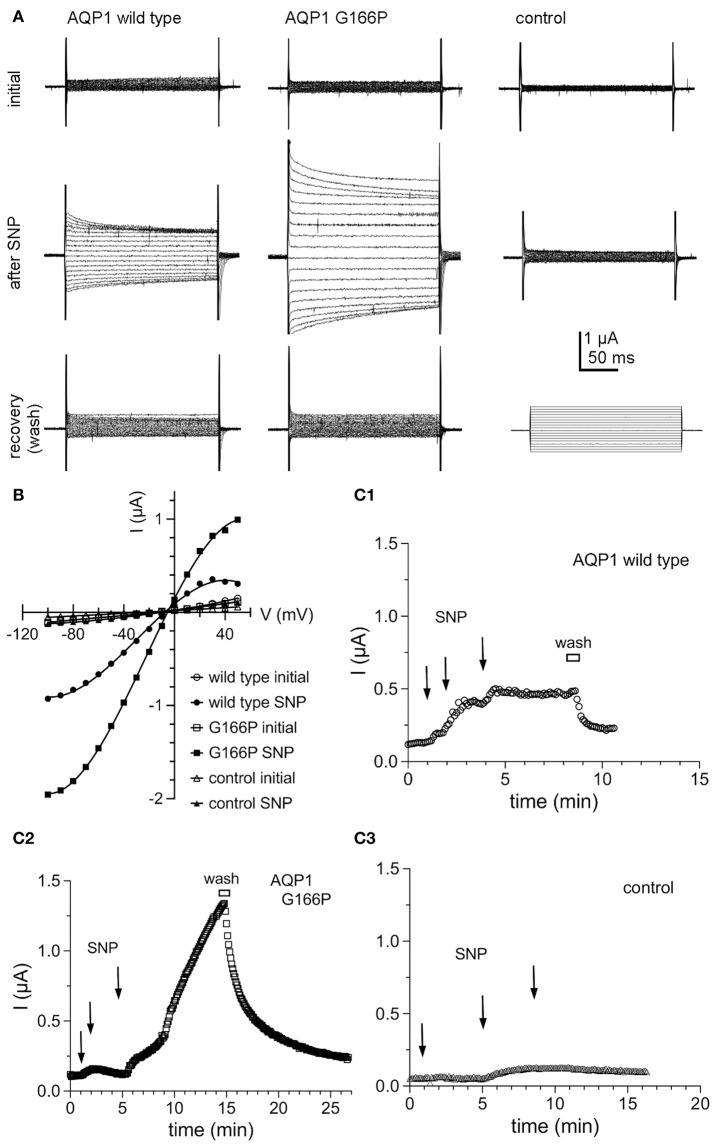
Ion conductance responses of oocytes expressing human AQP1 wild type and G166P channels. **(A)** Currents recorded from wild type (left), G166P (middle), and non-AQP expressing control oocytes (right) by two-electrode voltage clamp before (initial) and after stimulation of intracellular cyclic GMP by application of the nitric oxide donor, sodium nitroprusside at a final concentration of 7.5 mM (after SNP). Perfusion of fresh bath saline without SNP (wash) promoted rapid recovery. **(B)** Current voltage relationships for the traces shown in **(A)**. **(C)** Steady state current amplitudes at +40 mV monitored as a function of time after three sequential applications of SNP (2.5 mM each) at times indicated by arrows, and after perfusion of bath saline without SNP (wash) shown by the horizontal bar, for wild type **(C1)**, G166P **(C2)**, and control **(C3)** oocytes. Data are from the same oocytes as shown in **(A)**.

The conductance properties of AQP1 G166P-expressing oocytes, as compared with wild type and non-AQP control oocytes, are summarized in Figure 7. The ion conductance responses of AQP1 wild type and G166P-expressing oocytes showed an increase in amplitude but not in apparent kinetics (Figure 7A), reversal potential (Figure 7B), latency to activation, or reversibility of the responses after bath washout with fresh saline to remove SNP (Figure 7C). Control oocytes showed negligible responses to SNP (Figures 7A,B).

## Discussion

**Figure 8:**
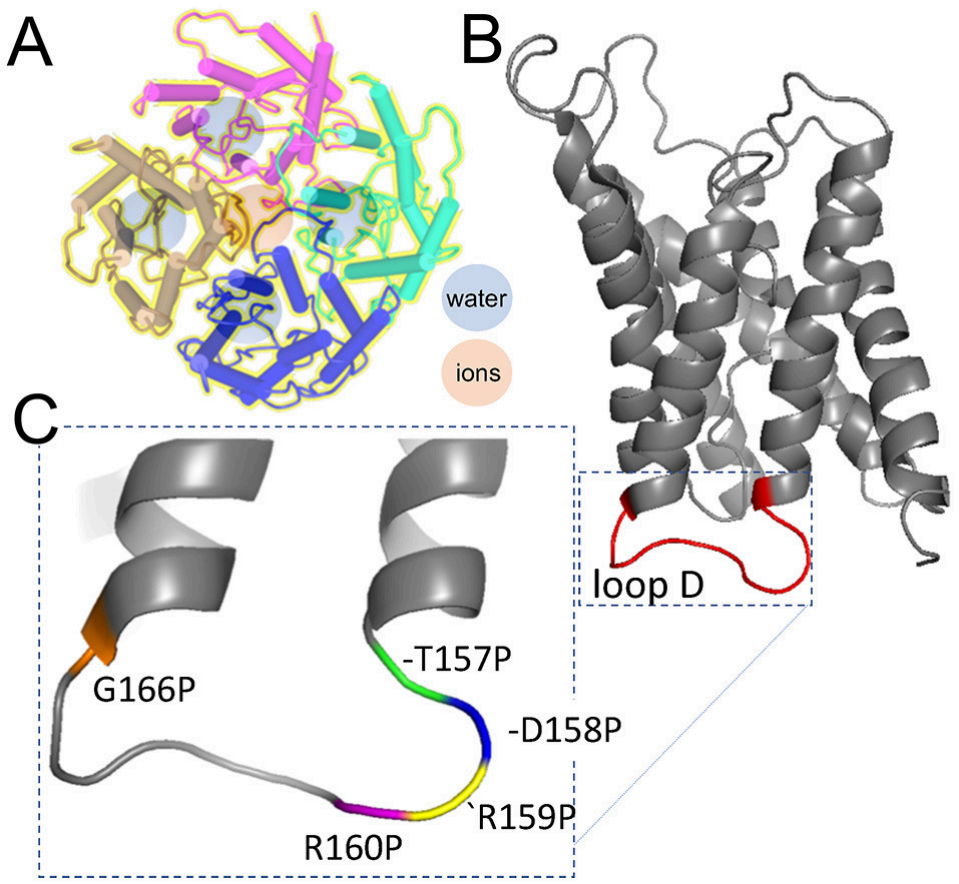
Schematic diagrams illustrating the separate pathways proposed to mediate water and ion transport in the AQP1 tetrameric channel, and the position of the mutations tested in the loop D domain by proline mutagenesis. **(A)** AQP1 channels assemble as homomeric tetramers in the membrane bilayer. Water pores (blue) are located in each subunit; cations are thought to permeate via the central pore at the four-fold axis of symmetry in the channel (rose). **(B)** Loop D is a cytoplasmic loop between the 4 and 5th transmembrane domains in each subunit; loops D in the tetramer surround the central pore. **(C)** Amino acid residues in loop D tested by mutation to proline. Structural data used to create the diagrams were downloaded from the NCBI Structure database (www.ncbi.nlm.nih.gov/structure/), for PDB ID 1IH5 human AQP1 (Ren et al., 2001); and PDB 1JN4 bovine AQP1 (Sui et al., 2001).

The aim of this study was to evaluate a candidate binding site for the AQP1 ion channel antagonist AqB011 suggested from prior *in silico* modeling, and to test the role of the intracellular loop D domain in AQP1 ion channel activation. Discovery of pharmacological tools for AQPs has been an area of keen interest for many years (Huber et al., 2012). As illustrated by the diagram in Figure 8, AQP1 ion channels are proposed to conduct solutes and water through pharmacologically distinct pathways (Saparov et al., 2001; Yool et al., 2002), with water transport mediated through the individual pores of the subunits (Jung et al., 1994), and ion transport proposed to be mediated by the central pore of the tetramer following activation by intracellular cGMP (Anthony et al., 2000; Yool and Weinstein, 2002; Campbell et al., 2012). The water channel function of hAQP1 is modulated by antagonists such as mercurial compounds (Preston et al., 1993); gold and silver compounds (Niemietz and Tyerman, 2002); the arylsulfonamide AqB013 (Migliati et al., 2009); medicinal herb compounds bacopasides I and II (Pei et al., 2016); aromatic carboxylic acid blockers referred to as CPD 1, 2, and 3 (Seeliger et al., 2013); and by agonist compounds such as AqF026 (Yool et al., 2013). Other inhibitors include TGN-020 for AQP4 (Igarashi et al., 2011), and gold-bipyridyl compounds for AQP3 (Martins et al., 2013; Graziani et al., 2017). The human AQP1 ion channel is pharmacologically distinct from the water pore, supporting the involvement of a separate pathway for ions through the central pore of the tetrameter (Figure 8). The AQP1 ion pore is blocked by Cd^2+^ (Boassa et al., 2006), other divalent cations (Kourghi et al., 2017b), and arylsulfonamide compounds AqB007 and AqB011 (Kourghi et al., 2016).

AqB011 inhibits the human AQP1 ion current but not the water flux, and slows the migration of AQP1-expressing human colon cancer cells (Kourghi et al., 2016). Molecular docking studies suggested that AqB011 might interact with a conserved arginine residues located on loop D domain of AQP1, a region that has been suggested to be involved in gating of the central pore of the AQP1 channel (Yu et al., 2006). The role of the conserved loop D domain was tested using a mutant construct of the AQP1 channel in which the positively charged arginine residues in positions 159 and 160 of the human AQP1 amino acid sequence were replaced with alanine. The mutation R159A+R160A did not prevent the channel from being expressed on the membrane of oocytes, as demonstrated by measured osmotic water permeability. The hAQP1 R159A+R160A channel had previously been thought to be non-functional as an ion channel (Yu et al., 2006). However, work here showed the R159A+R160A ion conductance was activated by CPT-cGMP albeit at a significantly slower rate, to a lower maximal amplitude, and with a longer latency than for AQP wild type channels, which would have made it difficult to detect in protocols used previously. Nonetheless the residual ion channel function in the R159A+R160A mutant was significantly greater than in non-AQP controls and was sufficient to allow evaluation of a possible difference in sensitivity to block by AqB011.

The ion conductance in wild type AQP1 expressing oocytes was significantly inhibited by AqB011, confirming prior work (Kourghi et al., 2016). In contrast, AqB011 had no effect on the ion conductance response in R159A+R160A expressing oocytes. These results provide evidence that AqB011 is acting directly on the AQP1 channel, and not indirectly through hypothetical native oocyte channels or transporters associated with AQP1 proteins. The plant AQP AtPIP2;1 is a dual ion and water channel which also is insensitive to AqB011 (Kourghi et al., 2017b). AtPIP2;1 has many amino acid sequence differences as compared to AQP1, but these include the absence of the poly-arginine series in loop D. Together these data suggest that selective pharmacological targeting of different classes of aquaporin ion channels will be possible, as structure-activity data for active agents continues to accrue, and discover of new agents expands the tools available for evaluating physiological roles of dual water and ion channels in the MIP family.

Proline scanning mutagenesis was used here to assess the role of the loop D domain in activation of the AQP1 ion conductance. Scanning mutagenesis is a method for analyzing the functional roles of amino acid residues in proteins by systematic replacement with another amino acid, such as alanine, cysteine, or proline (Cunningham and Wells, 1989; Kürz et al., 1995; Patel et al., 2013). Alanine is compact, lacking a bulky side group, and preserves 3D structure without influencing electrostatic characteristics (Cunningham and Wells, 1989). Alternatively, conformational structure can be deliberately altered by substituting residues with proline, which is distinctive in having the nitrogen atom covalently bound in a 5-membered ring, which impairs formation of intermolecular hydrogen bonds (Williams and Deber, 1991), and introduces “kinks” in secondary structure (Barlow and Thornton, 1988; Woolfson and Williams, 1990; Sankararamakrishnan and Vishveshwara, 1992). Proline scanning mutagenesis has been used to investigate gating mechanisms of ion channels such as the inward rectifier and transient receptor potential (TRP) channels (Sadja et al., 2001; Jin et al., 2002). Sadja et al. (2001) showed proline substitution in the second transmembrane domain of G-protein-coupled inwardly rectifying potassium channels shifted the channels into an active conformation, suggesting the site for Gβγ mediated gating. Dong et al. (2009) showed proline substitutions in the fifth transmembrane domain of TRPML1 ion channels locked the channels in an active state, which similarly allowed definition of the site of cation conductance gating. Proline scanning mutagenesis used here showed that the AQP1 cation channel is sensitive to mutations capable of altering the structure of the loop D domain, with both down- and upregulation of channel activity observed depending on the location of the mutation in the conserved amino acid sequence (Figure 8).

In sum, results here support the hypothesis that interaction of the inhibitor AqB011 depends on the structure of the loop D domain of the AQP1 channel, and that this domain is important for AQP1 ion channel gating. Aquaporin channels are more than simple pathways for the passive flux of water and glycerol. As a group they are increasingly being found to include highly specialized, regulated, multifunctional channels with diverse roles across the kingdoms of life (Gomes et al., 2009; Kourghi et al., 2017a). Results here contribute to understanding the structural basis for gating and pharmacological block of the human AQP1 ion channel, and add further evidence supporting the role of the central pore as the pathway for ion flux in human AQP1.

## Author contributions

MK, JP, and AY: Participated in the research design. MK, SN, JP, and AY: Conducted experiments and performed data analysis. MK, MD, and AY: Wrote the manuscript. MK, MD, SN, JP, and AY: Reviewed and edited the manuscript.

### Conflict of interest statement

The authors declare that the research was conducted in the absence of any commercial or financial relationships that could be construed as a potential conflict of interest.
